# Advancing policy design through creative engagement with lived experience: the Tomorrow Party

**DOI:** 10.1080/25741292.2024.2308311

**Published:** 2024-02-06

**Authors:** Michael Mintrom, Lisa Grocott, Shanti Sumartojo

**Affiliations:** Monash University, Melbourne, Australia

**Keywords:** Policy design, lived experience, imagined futures

## Abstract

We consider how lived experience might actively inform policy design. Good policy design calls for analysis of problems, how they might be addressed, and likely outcomes. Policy scholars and practitioners have devised methods that bring rigor to policy design through problem framing, assessment of potential interventions, and prediction of outcomes of those interventions. This pursuit of analytical and predictive rigor has often given short shrift to the insights of people whose lives are affected by current challenges and who will be impacted by policy change. Our theory of change is that creative engagement with citizens can generate insights of high value to the process of policy design. We introduce the Tomorrow Party – a design method for generating novel stakeholder insights regarding desirable future states. We then discuss initial findings from a series of pilots. Those findings suggest the Tomorrow Party is a broadly applicable creative tool for advancing policy design.

Our purpose here is to introduce The Tomorrow Party. This is a novel and creative method for engaging with people’s lived experience of social outcomes and generating new insights into the futures they value and desire. The Tomorrow Party asks participants to “time travel” to imagine a near-future where the policy changes they want are underway, and asks them how this future looks, feels and works. We found that generating improvised and dialogic “tomorrow stories” helps people work out the details of the changes required to get to the futures they want. It also helps build a sense of hope and commitment to reaching those futures.

Moreover, because participants can have a lived experience of the very policies they want to change, the Tomorrow Party addresses a perpetual tension in the making of public policy, which is that those who have most influence on policy design cluster in capital cities while those who live with the consequences of policy choices are broadly dispersed – often, across diverse settings. Jeffrey L. Pressman and Aaron Wildavsky, authors of the classic study of policy implementation (Pressman and Wildavsky 1973), recognized this tension and the programmatic dysfunctionalities that can arise because of it.

How might we increase the likelihood of policies being well designed? For those who have been trained in graduate public policy programs over the past few decades, the answer that most immediately springs to mind is that we need to promote rigor in the collection and analysis of relevant evidence of the impact of policy. That line of response has led to increasing sophistication in the deployment of data analytic methods and cost-benefit analysis. Treating public policies as investments and using actuarial methods to predict the return on investment represents a recent and vital contribution in this space (Mintrom 2019). It epitomizes the advancement of evidence-based policymaking. It holds the promise of more rigorous approaches to targeting and fine-tuning specific policy interventions. Such approaches to policy analysis and policy design can serve to identify those at most disadvantage in society and broadly assess the likely effectiveness of efforts to address that disadvantage. The approach underpins important developments like the growing recognition of the merits of early intervention to address social issues, the value of high-quality early childhood education, and the justice reinvestment movement.

But while this pursuit of data-based analytical rigor has proceeded, a more eclectic group of scholars and practitioners, heralding from a diversity of disciplinary backgrounds, have put forward a distinctly different set of responses concerning how we might increase the likelihood of policies being well-designed (Kimbell and Bailey 2017; Mintrom and Luetjens 2016; Van Buuren et al. 2020). These contributors often bring to policy design a sensitivity to lived experience and tend to focus on understanding the lives of people who must grapple with the consequences of policy choices (Doyle, Gardner, and Wells 2021; Durose and Richardson 2016; Isom and Balasuriya 2021). We show how we have done this through creative engagement, and introduce the Tomorrow Party as a new method we have prototyped in pilots with different stakeholders in the policymaking process.

The Tomorrow Party’s purpose is to promote further engagement of citizens in policymaking, through creative, speculative and dialogic means. The International Association for Public Participation (IAP2) has developed a Spectrum of Public Participation describing five public participation goals: “inform,” “consult,” “involve,” “collaborate,” and “empower” (IAP2 2018). Mapping the Tomorrow Party to that spectrum highlights its capacity to go well beyond “informing” and “consulting” stakeholders. The Tomorrow Party creates an opportunity for stakeholders to get involved, expressing their concerns and aspirations. It creates an opportunity to collaborate, contributing to the identification and development of alternative solutions to problems and, potentially, to indicate a preferred solution. The Tomorrow Party is not intended to empower in the way that IAP2 defines empowerment – that is, “to place final decision making in the hands of the public.” Yet, as we will elaborate, the Tomorrow Party certainly encourages people to speak up and actively contribute to policy discussions. Our contention is that engaging with a broad range of professional and personal experiences can surface new insights into not just what is needed but also what is longed for that policy can help to answer. Such engagement can create rapport amongst the communities who share an interest in policy outcomes. And it can engender a sense of agency in meeting those policy challenges.

While analytic, actuarial methods privilege quantitative data and positivist epistemic beliefs, the Tomorrow Party plays with design ethnography and creative futuring methods that respect subjective, co-constructed versions of what is possible. We take the view that these qualitative and speculative insights are just as necessary as quantitative approaches to policy analysis and development. Indeed, these divergent methods can be complementary in supporting improved policy design. The more we listen to the hopes and dreams of those who could benefit from specific policy interventions, the greater the likelihood that policy design will promote better social outcomes.

## 1. Design in the policy process

For decades, policy scholars and practitioners have employed the term “policy design” to describe core elements of their practice of transforming policy analysis into proposals for policy change (Bobrow and Dryzek 1987; Howlett 2011; Linder and Peters 1991). In so doing, they have revealed a predilection toward more formal application of design theory and practice to inform the practice of policy design (Schön 1992). Thus, policy scholars quickly took note when scholars and practitioners in fields proximate to policy studies espoused the merits of emulating professional design practice when designing organizational processes and systems (Brown 2009; Liedtka, King, and Bennett 2013). Various initial efforts were made to document the ways that design theory could explicitly inform aspects of the policymaking process (Howlett 2014; Mintrom and Luetjens 2016). Today, it is increasingly understood within policy studies that explicit application of design theory has the potential to improve problem definition and mechanism design (Bason and Austin 2022). Activities informed by design theory can support policy analysts and policy designers to better understand how citizens experience challenges in their local settings and what government services would best support them in addressing and overcoming those challenges. Moving forward, we expect design will inform all stages of the policymaking process: problem definition, mechanism design, options assessment, agenda setting, policy adoption, implementation and evaluation.

Design in the policy process is at an exciting juncture. We now see a variety of approaches that come directly from design thinking processes, co-design practices and design theory being deployed by policy analysts and designers. These approaches tend to bring together design approaches with methods from other fields. Design thinking has been explored as an innovative process for developing policy, with design-informed policy labs like the UK’s Policy Lab and New York City’s Public Policy Lab putting these ideas into practice (McGann, Blomkamp, and Lewis 2018). Critical design theory complements this work by questioning how to facilitate participatory relationships with community, navigate the uncertainty of future making (Pink, Akama, and Sumartojo 2018), respect relational co-design commitments (Akama, Hagen, and Whaanga-Schollum 2019) and engage with anticipation and the not-yet-known (Korsmeyer, Light, and Grocott 2021). Service design articulates the value of deliberately attending to the human touchpoints by mapping the “journeys” that clients take as they negotiate various government services (Radnor et al. 2014; Penin 2018). Most relevant to a discussion about lived experience are the methods and epistemic position of co-design or participatory design and the value they place on plural perspectives, participatory exchanges and a social commitment to designing more just futures (Blomkamp 2018). As knowledge expands of such explicit use of design methods in policy processes, understanding is growing of the role creativity plays in anticipating and storying preferred futures.

## 2. Emerging interest in lived experience

Just as elements of design theory have recently received increased attention from policy developers, increasing efforts are now being made to inform policy design with insights from individuals with lived experience of circumstances requiring some form of government intervention or assistance. See, for example, the mental health lived experience engagement framework developed by the Victorian Department of Health and Human Services in Australia (2019) and the World Health Organization’s framework for meaningful engagement of people living with noncommunicable diseases and mental health and neurological conditions (2023). The term “lived experience” refers to the unique and subjective understandings that individuals gains through their personal life experiences, encompassing the physical, emotional, psychological, and social experiences that shape people’s worldviews, perspectives and aspirations. Contributors to qualitative research traditions have long recognized the value of tapping the lived experiences of individuals and communities to document and understand a range of social processes and the variety of impacts they can have (Ellis and Flaherty 1992; Simey 1961). The claim of researchers contributing to such work is that much has been missed by social research that seeks to understand the human experience as a series of rational, cognitive choices and actions that can be encapsulated and summarized in quantitative measures. To provide a fuller understanding of the human condition, researchers have drawn on various methods including ethnography (Hammersley and Atkinson 2019; Atkinson et al. 2007) and auto-ethnography (Adams, Jones, and Ellis 2022; Denshire 2014). In the process, they have been able to document life experiences where the profound is found hiding in the everyday. Parallel to qualitative researchers increasingly seeking to document and give validation to the importance of lived experience, advocates for groups that have historically been subject to exclusion from decisions affecting their lives have been calling for change. The mantra of “nothing about us without us” (Stack and McDonald 2014) neatly encapsulates the claim for marginalized voices to be heard and to have influence within policymaking processes.

Evidence from lived experience has the potential to advance human flourishing through improved policy and program design. But inevitably, the voices of lived experience must compete for influence in a crowded field of policy advocacy. Any review of influential theories of policymaking rapidly reveals that policymaking has long been and remains the domain of professional and social elites (Weible 2023). While policy settings can have profound and differential impacts on the life chances of individuals and groups, opportunities are limited for many people outside of established policymaking circles to have their voices heard and to influence policy design in meaningful ways. This raises the question of how those with relevant lived experience might contribute to efforts to improve policy design. One way is through the provision of testimony during policy inquiries and consultation processes (Mintrom, O’Neill, and O’Connor 2021). However, thoughtful consideration is needed when engaging with lived experience to ensure the methods are not reductive or extractive and to reduce the risk of further causing harm in the process of asking people to share their story (Skelton-Wilson et al. 2021). As an interdisciplinary team of researchers, we question how policy design might explore less extractive, more co-creative approaches to learning from peoples’ lived experience. Specifically, we are interested in how processes of policy design might move from inviting people with lived experience to *report on their past* to more speculatively engaging them to *imagine futures*.

## 3. The Tomorrow Party

We developed the Tomorrow Party to explore and analyze how a lived experience activity set in the future might broaden participation in processes of policy design and offer different types of “evidence” to those processes. This imaginative and playful activity invites participants to travel forward in time and share stories in the present tense of the desirable future they are living in. As a participatory story-making process, the Tomorrow Party generates novel ways of sharing plural perspectives on possible futures so we can collectively anticipate what is at stake and work out what policy responses would contribute to the futures we want.

Many methods have been developed to promote futures thinking. The Tomorrow Party aligns with approaches that Muiderman et al. (2020) have collectively termed “pluralistic futures, societal mobilization and co-creating alternatives” (8). Distinct from futures methods that rely on predictive modeling or participatory envisioning activities that give voice to a range of perspectives, the Tomorrow Party calls for individuals making sense together of a not-yet realized future. Participants are not strategically looking at a future from above, or calculatedly predicting from afar. Instead, the centering of lived experience asks guests at the Tomorrow Party to be themselves at a party five years in the future. This first-person future perspective allows guests to discover what is valued and needed in this future by improvisationally storying what it looks and feels like to live in this imagined yet desirable world. Critically, the futures-orientation allows for policy insights and ideas that ensure understandings of lived experience are not fixed to a moment in time, or that efforts to envision possible futures are not curtailed by lack of imagination.

### 3.1. Designing the Tomorrow Party

In creating the Tomorrow Party, we sought to better understand how to design an encounter that worked for the guests and the hosts. We closely attended to how the experience of story making and time traveling advanced the guests’ personal or professional commitments, deepened their perception of the work ahead and their capacity to seed change. Similarly, we paid attention to how we might generate and record tomorrow stories that could be later analyzed and shared to inform policymaking. This allowed us to explore and address three core questions.

First, we asked: How does the party work for guests? We were interested in what prompts and props best support people to engage their imaginations and share their experiential stories and creative ideas to generate useful insights for future policy. For example, asking people to dress for a party, to hold drinks or snacks, or to bring a party invitation that we have sent them can all help to set the tone of the activity. However, we sought to steer away from props that might distract from the speculative goals or make it harder for people to suspend disbelief and invest in the encounter. Our paramount concern was the focus on care and how to design the atmosphere as much as the smooth facilitation of the tasks.

Second, we asked: How and when do we engage different stakeholders respectfully in the process, mindful of the ethical obligations associated with engaging people’s memories and dreams? We were conscious of the diversity of lived experiences that can be found in a given community, or amongst policymakers, and knew that there will be times when people are ready to share their own experiences as part of engaging in future speculation and times when they are not. We also wanted to explore the upper and lower limits of participant numbers.

### 3.2. Methodology for understanding the Tomorrow Party

We also asked: How can we ensure that insights from the Party conversations can help inform policy development? We took a design ethnography approach (Pink et al. 2022), working within the format of a designed workshop encounter to ask participants to embrace future uncertainty and generate speculative “tomorrow stories” in dialogue with each other. Indeed, we participated in the parties ourselves, prompting each others’ stories and helping new ones to emerge amongst participants.

Our analysis of these stories was made in two ways: We analyzed our own experiences dialogically, through continually discussion of our own experiences and insights, a process described as dialogic team auto-ethnography (Sumartojo, Edensor, and Pink 2019). In addition, during the party, we made audio recordings of the tomorrow stories at their final phase, videoed and took photographs of the party in action, and asked participants to write down or video their responses to the party at its close. By using both text-based and audio-video methods for recording activities, we made research materials that we could then analyze to make sense of how people thought and felt about the future and the Party’s potential as a tool for creative engagement with policymaking. We also conducted some interviews after the Party, when the experience had time to sink in, and asked people what they learned from attending the event and what its value as a method might be. These research materials were shared with our Monash University-based research partners in Fire to Flourish, who could then analyze the tomorrow stories and identify the best ways for them to influence policy alongside their other advocacy work. In this sense, the contribution of the Tomorrow Party to policy development recognizes the importance of close partnerships between researchers and other bodies, and relies on the expertise of partners to discern the most effective input into policy.

### 3.3. Running the Tomorrow Party

The Tomorrow Party research project included three pilots where we analyzed and evaluated the stories and the guests’ experience. We also held seven additional parties that allowed us to iteratively refine the party design and facilitation. In all three pilots, we worked with between 20 and 40 party guests, but the additional parties experimented with 100+ guests, online, and intimate groups. We adopted an approach to hosting that sought to welcome and engage personally with as many participants as we could as facilitators, and an iterative approach to refining each pilot. The three pilots took place in Australia and the other parties took place at conferences, research meetings and specially convened events in Australia, Denmark, the UK, New Zealand and Dubai.

The first pilot was a party in Melbourne with policy makers, policy researchers, and project advisors. The goal was to hear from policymakers tomorrow stories about a future where approaches to policymaking directly integrated insights from lived experience. This was also an opportunity to refine our facilitation practices so relevant insights could be generated. Our research partner Fire to Flourish, was the co-host for the second party during the Australian Disaster Resilience Conference 2023 in Brisbane. Working at the intersection of disaster resilience and community development the party guests included policy designers, disaster response professionals, community leaders and representatives of areas affected by Australia’s 2019/2020 wildfire season. To ensure insightful stories could emerge from this diverse group of stakeholders, our goal was to learn, from hosting a party, how to create an atmosphere of trust, how to scaffold peoples’ capacity to imagine a near future, and how to support participants to become united around a shared sense of purpose. The third pilot brought together policy designers, social innovators and co-designers in Melbourne who we anticipated could help us co-analyze the potential of the Tomorrow Party in relation to community engagement and facilitation practices.

We initially assumed the Tomorrow Party method would be most useful at the insight-gathering phase of policy development. However, we learned it also has potential, with different prompts, to be utilized at different stages in the policy process – mechanism design, options assessment, agenda setting, implementation and evaluation. We heard from guests how the party was effective in building rapport between potential partners, hope for what is possible, and a commitment to take future action.

In prototyping the Tomorrow Party, we did not assume that people with lived experience exist outside of the system designing for change, or that any one person’s experience can be deftly fixed in time. From the outset, the Tomorrow Party was informed by Indigenous conceptions of non-linear time and guided by principles of relationality, responsibility and respect for the participants (Yunkaporta 2019; Simpson 2017). Critically, the social exchange is grounded in reciprocity. To minimize the harm an extractive process can wield, the party creates a convivial atmosphere where the role of researcher is reframed as host and the research “subjects” are guests. In this way, the tomorrow stories generated are as much for the community co-creating them as they are for surfacing policy insights. This framing allowed the act of storying an adjacent possible future to be an agential experience for the guests and an illuminating one for the researchers. Our motivating theory of change was that the efforts to *creatively* engage with peoples’ expertise and lived experiences can generate highly contextual insights that can be of high value to the policy design process. We developed the Tomorrow Party to serve as an adaptive and responsible creative tool for sense-making from an emergent future about what really matters to people. Our initial focus was on gathering tomorrow stories to guide the development of appropriate policy responses to various existing and emerging policy challenges. We soon came to understand that, beyond policy insights, the stories from the future also deepened the potential for social impact by way of forging strategic partnerships and building a community’s capacity to imagine more just futures.

The Tomorrow Party, as a research project, sought to explore how stories of an imagined future lived experience might creatively reveal the preconditions necessary to transition from the current to the desired future state. By meaningfully reframing someone’s lived experience as not tethered to the past but grounded in how they perceive the world, we expanded the potential for lived experience insights to include ideas for creating desired futures that participants hold dear or have the potential to imagine. The Tomorrow Party is intended to enable empathic exchanges that do not hide the complexity of the future situations, but instead enact respect and shared responsibility for reaching a mutually-desired future. Similarly, Indigenous orientations to folding time offer ways to think of the future as residing in the present and the imagined future advocating to become the past. This orientation to time asks us to respect lived experience as something that is always shifting, embodied and relational.

Through conducting these pilots, our goal was to clarify the contribution of the Tomorrow Party. Our most basic evaluation question was: To what extent is this activity attaining its goals? To understand this, we deliberately reflected together after each pilot to identify what we had learned and how we could best apply these learnings in future pilots. Our qualitative evaluation approach drew on design ethnographic techniques that are well-established for understanding how design interventions can learn about lived experience (i.e., Sanders and Stappers 2014; Pink et al. 2022). Using a range of written and audio-visual research materials, we explored the perspectives, experiences, and stories of Tomorrow Party participants to understand Party processes and insights from their viewpoints. Throughout, we sought to triangulate across forms of evidence gathered during and after each party to build a clear picture of what had happened, what went well, what could be improved, and how we could improve on our practice in subsequent pilots.

The Tomorrow Party relies critically on facilitating and enabling lively, creative interactions among a diversity of participants. The creative catalyst in the Tomorrow Party is the permission it gives to break free from typical ways of thinking about community challenges, while not requiring participants to learn a process of innovation that may have little relevance to their lived experience. The Party invites everyone to engage in a way that might be novel in a professional setting, yet is familiar in a social context. People chattering in the present tense about how their lives have been changed by not-yet-imagined policies creates a space of possibility. The back-and-forth conversational structure makes space for the party-goers to reflect on the specific qualities of the future world. Because they take place at a fictional future event, these animated conversations intentionally locate policy shifts in the affective realm and in the everyday details of life. In coloring the world with details of how they feel, what it looks like, and how the world has changed, it becomes clearer where people want to be and how they might get there. Staying in a generative place of suspended disbelief, it becomes possible to resist falling into all the reasons why something cannot happen. In this way, default cognitive modes of critique, analysis, or problem-solving can be replaced with the affirmations of improvizational play that open up important forms of connection, agency and commitment to action.

## 4. Initial findings

We have drawn four initial findings from the Tomorrow Party pilots. First, the Tomorrow Party opens space for creative exploration. Second, it serves as a vehicle to move from recounting of lived experience to scoping out imagined futures. Third, it provides a venue for sharing pre-figurative practices. Fourth, it opens the possibility of artifacts emerging from imaginary play being deployed to support subsequent policy advocacy work. These findings are by no means exhaustive. Yet, together, they indicate the opportunities for learning that the Tomorrow Party affords. Here, we discuss each in turn.

### 4.1. Opening space for creative exploration

The Tomorrow Party, like all good parties, establishes an atmosphere of lightness and combines this with imagined time-travel to the future. This opens space for creative exploration. As a result, we have seen party guests give voice to possibilities they would feel uncomfortable sharing in the formal atmosphere constructed by a boardroom meeting. As one guest gives voice to an apparently audacious possibility, they implicitly give permission to others to do the same. The result can be a vibrant exploration of future scenarios, where the attention of guests can bracket the constraints of the present to alternatively build hope for an emerging future. Much of what comes from these excited discussions of the future might seem improbable. Yet, repeatedly the guests affirmed that giving voice to a not-yet future was liberating and energizing and rapidly built shared purpose and rapport and oftentimes empowerment. Further, we found the goodwill toward the people you are time traveling with and the sense of possibility co-created in a Tomorrow Party extends beyond the duration of the gathering, smoothing the way for concrete explorations of futures that, in the absence of a Tomorrow Party, might not have been considered at all.

### 4.2. Moving from lived experience to imagined futures

The growing interest in lived experience informing policy reviews and policy development initiatives is premised on the expectation that voices of experience will counter entrenched, elite dominance of policy discussions. Yet a risk remains that sharing of lived experience – which can often be retraumatising – does not serve to recalibrate policy design efforts. Encouraging the sharing of lived experience is different from inviting those with lived experience to participate in policy design work. The Tomorrow Party provides a vehicle through which people with relevant lived experience can find their voices and, in the process, express aspirations for the future that might have otherwise not been heard. We repeatedly heard how the Tomorrow Party can make people feel safe and supported in expressing their aspirations for the future.

The time-shift is critical. While lived experience is typically shared with a backwards gaze, the Tomorrow Party invites participants to talk excitedly in the present tense of their imagined *future* state. The party atmosphere encourages those with lived experience to envision and extrapolate, to creatively engage with past experience to imagine future possibilities. We have observed that time-shift to be cathartic for people with lived experience because it gives them a sense of their own agency in setting the terms of a future state. The time-shift is also empowering because it takes participants beyond the static recounting of lived experience. They enter a space where lived experience becomes a springboard for creative exploration of future possibilities.

### 4.3. Sharing pre-figurative practices

The Tomorrow Party opens opportunities for imagining future possibilities. Prompts encourage conversation that is playful and speculative in nature. Thus, the party can serve as a venue where flashes of inspiration emerge through dialogue among participants. While there is a deliberate effort to have participants immerse themselves in an imagined future, it is inevitable that their prior experiences and knowledge will anchor aspects of their imaginings. We see this as desirable, because this allows lived experience to organically inform discussions of how the future might be. Through our Tomorrow Party pilots, we have also observed some interesting and potentially highly fruitful sharing of pre-figurative practices.^1^ For example, as they construct a future scenario together, participants often bring to the conversation reflections on their current practices and how elements of those practices might shape future practices. This is where the flashes of inspiration emerging through dialogue can promote new ways of perceiving what current practices work well, which ones do not, and what adjustments would be desirable moving forward. The anchoring and adjustment taking place through dialogue about the future can generate ways of appreciating what aspects of the present situation could usefully serve to support better future outcomes, and how they could or should be changed. All of this could be equated with the incrementalism and partisan mutual adjustments characteristic of traditional policy development work (Lindblom 1959), but instead of analyzing how this has happened in the past, the Tomorrow Party casts forward, folding the present and the future together. The party atmosphere gives license for audacious creativity and encourages participants to take inspiration from one another in ways that deliberately shift the focus from the present. In that sense, the building from current practice is much more playful and serendipitous than the “muddling through” of traditional policy design work, or the forensic analysis of policy “lesson drawing” (Rose 1993).

### 4.4. Building artefacts of imaginary play into advocacy work

Over the three Tomorrow Party pilots, we introduced different ways of creating materials that reflected people’s insights during imaginary time travel. For example, reflecting on the ways that thoughts often come to us after a meeting or a conversation, we planned to invite participants to share with us their “driving home” thoughts - this gradually developed into a “guest book” where people shared their thoughts as they left, and a “thank you letter” sent by e-mail around two weeks after the party that asked how they might still be thinking about it. These provided additional perspectives on what had been shared in the party setting and the implications of specific elements of the party conversations.

Additionally, we introduced time for toasts at the end of the party. These were conducted like traditional party toasts, with the twist that speakers were asked to reflect on lessons *from* the future, as opposed to giving summary lessons *for* the future. These contributions were documented using video, audio recordings, or writing samples. Through the process of collation, we came to see that some artifacts of imaginary play held the potential to be redeployed in policy advocacy work.

While use of these prompts and artifacts in this manner would go beyond the scope of our project, we see future possibilities. Foremost, we view the Tomorrow Party as a mechanism for contributing new insights into policy design work. But we now also understand that artifacts generated in Tomorrow Parties could support advocacy regarding specific policy options. We anticipate many fragments of conversation and imagining generated in Tomorrow Party settings could contribute in innovative and compelling ways to policy advocacy work beyond the design stage of policy development. We should also add that the spontaneous goodwill generated in each of the Tomorrow Party pilots was extraordinary to observe. We are confident that such goodwill also represents a residual artifact of the party. Building on insights from the Advocacy Coalition Framework (Nohrstedt et al. 2023; Sabatier 1988), we speculate that this goodwill could be of significant value elsewhere in the process of policymaking, such as advocacy and agenda setting.

## 5. Conclusion

Policy scholars and practitioners have produced various methods that bring rigor to policy design, through problem framing, assessment of potential policy interventions, and prediction of how well such interventions will generate valued outcomes. This pursuit of analytical rigor has tended to give short shrift to the perspectives and creative insights of people whose lives are affected by current challenges and who will be impacted by policy change. As a corrective to that, and in response to the growing call for lived experience to inform policy development, we prototyped and tested the Tomorrow Party and explored how it might inform policy design. Our theory of change was that efforts to creatively engage with citizens can generate insights of high value to the policy design process. The Tomorrow Party surfaces the concerns, hopes and aspirations of people with diverse but relevant perspectives, and explores how these might be best brought together and incorporated into policymaking processes. We invited citizens, policy designers, community facilitators and researchers to listen, empathize, reflect and learn from each other in a speculative and playful way. In the process, we saw how a diverse range of expertise might inform policymaking processes and how bringing a care-based approach to the design of each phase of the party creates a generous, compassionate atmosphere for sharing deep insights from policy-related conversations. These type of highly contextualized, place-based insights can be easily missed in conventional forms of stakeholder consultation that resist engaging with people’s felt and lived experiences. The Tomorrow Party presents a way of navigating toward better policy development processes that might take us closer to desirable future states.

The findings presented here suggest how future iterations of the Tomorrow Party could be arranged to further explore practical connections between creative engagement, policy design, agenda setting and policy advocacy. These findings suggest the Tomorrow Party is a flexible, contextual, and innovative method for policy design to learn from lived experience, surface assumptions, develop future thinking capabilities and nurture resonant policy narratives. It represents a broadly applicable creative tool for advancing policy development in a range of policy and community contexts. Indeed, like other contributions using design theory to inform policy development, the Tomorrow Party could be applied elsewhere in the policy process, including to support aspects of agenda setting, implementation and evaluation.

## Note

1 Our use of the term “pre-figurative” is borrowed from Sheila Rowbotham’s writings on feminism. In using the term, Rowbotham highlighted the transformative potential of social movements and the need to create spaces and practices that challenge existing power dynamics and envision alternative ways of living and organizing society. See, e.g., her contributions in Rowbothan and Mitter (1994).
